# No Correlation Between Mood or Motivation and the Processing of Global and Local Information: Online Replications of Gable and Harmon-Jones (2008) and Domachowska et al. (2016)

**DOI:** 10.1027/1618-3169/a000562

**Published:** 2023-01-19

**Authors:** Alberto De Luca, Stephan Verschoor, Bernhard Hommel

**Affiliations:** ^1^ Cognitive Psychology Unit, Institute of Psychology, Leiden University, Leiden, The Netherlands; ^2^ Cognitive Systems Lab, Mathematics & Computer Science, Bremen University, Bremen, Germany; ^3^ Cognitive Neurophysiology, Department of Child and Adolescent Psychiatry, Faculty of Medicine, TU Dresden, Dresden, Germany; ^4^ Cognitive Psychology, Faculty of Psychology, Shandong Normal University, Jinan, People's Republic of China

**Keywords:** motivation, attention, mood, global-local processing

## Abstract

**Abstract.** Mood has been argued to impact the breadth of human attention, but the empirical evidence supporting this claim remains shaky. Gable and Harmon-Jones (2008) have attributed previous empirical inconsistencies regarding the effect of mood on attentional breath to a critical role of approach/avoidance motivation. They demonstrated that the combination of positive affect with high, but not with low, motivational intensity improves performance during processing local information and impairs performance during processing global information. The latter, but not the former, was replicated by Domachowska et al. (2016). Since we were interested in the modulation of attention by valence and motivation, and considering the inconsistencies in the findings, we replicated the critical experiments of both studies in four online experiments but found no significant effect of either valence or motivational intensity on attention. Taken together, our evidence casts doubt on a systematic relationship between mood or motivation on the one hand and global/local processing on the other.


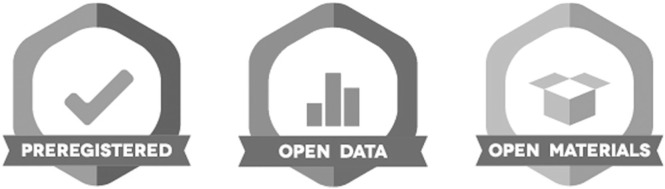


## Public Significance Statement

Mood is assumed to color the way we perceive and attend to our environment, but the evidence supporting this assumption is shaky. Here, we try to replicate two previous demonstrations of how mood and motivation affect the kind of visual information we process, but without any success. This raises doubt about the idea that mood directly impacts visual attention.

## Introduction

The way people process information is strongly influenced by emotions and moods (Bolte & Goschke, 2009; Goschke & Bolte, 2014). For instance, several recent studies have demonstrated a decisive role of positive mood in coping with stress, trauma, and adverse life circumstances, with considerable implications for intervention (Fredrickson et al., 2003; e.g., Fredrickson, 2001), and mood has been shown to improve various kinds of divergent thinking in solving creativity tasks (Baas et al., 2008; Isen et al., 1987), including idea generation (Mastria et al., 2019). These findings have been generally interpreted as evidence for the idea that positive mood is associated with increased cognitive flexibility (Dreisbach & Goschke, 2004) and a broadened focus of internal and/or external attention (Fredrickson & Branigan, 2005), which promotes the consideration of a broader range of environmental stimuli and the activation of a more extended network of thoughts and memories (Lee & Sternthal, 1999; Nadler et al., 2010). This widening of the attentional focus benefits tasks that rely on the integration of information but tends to impair performance in tasks requiring focused attention and filtering out distracting information (Bolte & Goschke, 2009; Goschke & Bolte, 2014; Zwosta et al., 2013).

Such a scenario would fit with the implications of the theory of metacontrol (Hommel, 2015). This theory claims that the way humans process information is modulated by a mechanism that puts them in either a more flexible state or a more persistent processing state. Given that mood has been shown to influence metacontrol, with more positive mood resulting in a less focused, more integrative metacontrol state (for a review, see Hommel & Colzato, 2017), it would fit if such less focused, more integrative states would come with a widened attentional focus. Given our recent studies on the relationship between metacontrol and attentional focus (De Luca et al., 2022), we, therefore, wondered whether their shared sensitivity to mood might provide a theoretical bridge between metacontrol and attentional focus.

While it is clear that mood can have effects on attention, it is less clear by means of which mechanisms it achieves them. More specifically, the so-far strongest claim of a direct connection between mood and attention, the GLOMOsys model of Förster and Dannenberg (2010), does not provide any mechanistic consideration regarding how such a connection might operate. Moreover, recent studies have raised doubts about the replicability of the empirical basis of GLOMOsys (e.g., Field et al., 2016; Ijzerman et al., 2020; Klauer & Singmann, 2015; Reinhard, 2015). A possible reason for the shaky empirical basis of mood effects on attention might be that important moderators of the effect have been neglected or not yet fully understood, thus rendering experimental manipulations of mood ineffective. Indeed, Gable and Harmon-Jones (2008) argued that previous studies investigating how emotions affect attention used a variety of affect-inducing manipulations (e.g., watching funny movies, recalling pleasant memories, receiving a small gift) that confound emotional valence with motivation. They concluded that previous studies left the possibility open that the broadening effects of positive mood on attention might have been the result of low motivational intensity rather than positive valence.

In Experiment 2 of their seminal 2008 study, which is of particular importance for our considerations, Gable and Harmon-Jones assigned participants to a positive mood induction condition (through pictures of delicious food) or a neutral mood induction condition (pictures of rocks) to manipulate valence. However, the particular impact of valence on the processing of global and local information was assumed to depend on motivational intensity. Positive affect low in (approach) motivational intensity (e.g., delight) was thought to signal that goal pursuit goes smoothly or that the action goal has been accomplished, indicating no need for effortful control and encouraging an exploratory processing style with a broadened attentional focus. In other words, low motivational intensity should promote the processing of global information. In contrast, positive affect high in (approach) motivational intensity (e.g., as induced by pictures of sweet food that elicit an appetitive motivational state connected with the goal to eat) was assumed to signal a possible new goal, which in turn would lead to a narrower breadth of attention and facilitate the processing of local, rather than global information. Indeed, Gable and Harmon-Jones (2008) found evidence of a narrower attentional focus (i.e., impaired performance on global and improved performance on local stimulus features) when participants were presented with positive stimuli high in motivational intensity compared to neutral stimuli or positive stimuli with low in motivational intensity.

The motivational dimensional model of affect (Gable & Harmon-Jones, 2010b), based on findings like the above, distinguishes between positive versus negative valence on the one hand and intensity of approach versus avoidance motivation on the other, and it claims that motivation, but not mood, is responsible for effects on global/local information processing. This idea was further corroborated by showing that in the Navon letter task, negative affect low in motivational intensity (induced by sad pictures) led to an increase in attentional breadth, whereas negative affects high in motivational intensity (disgust) caused a narrowing of attention (Gable & Harmon-Jones, 2010a). Additionally, Liu et al. (2014) reported further evidence that motivational intensity modifies the effect of positive affect on attentional flexibility during an attentional switching task, where positive-low affect increased cognitive flexibility at the expense of increased distractibility, whereas positive-high affect reduced distractibility but increased perseverance. Of particular importance, and in contrast to GLOMOsys, the dimensional model thus postulates that the attentional breadth and focus on global or local stimuli do not depend on the valence of mood or mood-related stimuli but on the motivational intensity they induce. Accordingly, strong motivational stimuli are assumed to narrow the focus of attention (and improve local processing) and weak motivational stimuli to broaden the focus (and improve global processing), regardless of whether their emotional valence is positive or negative.

Although the motivational dimensional model of affect introduced by Gable and Harmon-Jones (2010b) has been influential in recent research on emotion and attention (see Yang et al., 2022, for a review), there are several inconsistencies in the empirical findings. For instance, a study by Friedman and Förster (2000) found that priming approach motivation (by instructing participants to flex their arm) broadened attention focus, while priming avoidance motivation narrowed it. Friedman and Förster (2001) performed another experiment in which the participants’ task was to lead a mouse out of a maze to find a cheese piece (approach condition) or escape a hovering owl (avoidance condition). After the approach condition, a Navon task (Navon, 1977) exhibited broader focus of attention, while the avoidance condition resulted in a narrower focus. Accordingly, the motivational dimensional model (Harmon-Jones et al., 2012) does not predict the effects of avoidance conditions on motivational intensity observed in studies of Friedman and Förster (2000, 2001). Additionally, Baumann and Kuhl (2005) found that positive affect may have different effects depending on whether participants currently engage in global or local processing modes. Moreover, Huntsinger (2012) found that positive mood is a means of strengthening the dominant mode rather than moderating it. Finally, some studies failed to replicate the effects of positive mood on attentional focus (Bruyneel et al., 2013).

In light of the theoretical importance of the motivational dimensional model, the inconsistent evidence, and the fact that most replications of the findings by Gable and Harmon-Jones (2008) came from the same research group, Domachowska et al. (2016) conducted a direct (Experiment 1) and a conceptual (Experiment 2) replication of Experiment 4 of Gable and Harmon-Jones (2008). In the direct replication, they used the original stimuli and setup, whereas in the conceptual replication they used stimuli adjusted for their German participants to control for cultural differences. Additionally, to directly compare positive high affect with positive-low affect and an additional neutral condition, they changed the design into a 3 (positive-high affect vs. positive-low affect vs. neutral affect) × 2 (local vs. global targets) within-subjects design. They used the same logic as the original Experiment 2 of Gable and Harmon-Jones (2008), selecting mouth-watering food pictures for high-positive stimuli, animal and flower pictures for low-positive ones, and additional pictures of objects with lower valence for the neutral condition. The list of the stimuli and their respective ratings can be found in the supplemental materials (De Luca, 2022, Appendix A). Although Domachowska et al. (2016) argue that their “results increase confidence in the generalizability of the original findings across cultures, as well as across different stimuli” (p. 50), both replication attempts could actually replicate only half of the original observations: While Domachowska et al. (2016) successfully reproduced the impaired global processing with positive pictures high in motivational intensity reported by Gable and Harmon-Jones (2008, Experiment 2), they did not find any evidence for the improvement of local processing in the positive/high condition. Moreover, the comparison of attentional focus between the condition with positive stimuli of high motivational intensity and with positive-low motivational intensity failed to replicate the narrower focus for the positive stimuli of high motivational intensity as reported for Experiment 1 by Gable and Harmon-Jones (2008).

To summarize, it remains unclear whether positive mood has any systematic effect on processing global and local stimulus features. While motivational intensity has been taken into account for the empirical inconsistencies, the empirical basis for this assumption is weak. Accordingly, before exploring the relationship between the motivational dimensional model of affect (Gable & Harmon-Jones, 2010b) and metacontrol (Hommel, 2015), we were interested to see whether the underlying empirical phenomena pertaining to the modulation of attention by mood and motivation are replicable in the first place. Accordingly, we decided to run well-powered conceptual replications of Experiment 2 of Gable and Harmon-Jones (2008) and Experiment 2 of Domachowska et al. (2016) in two online experiments (Experiments 1A and 1B, respectively). To assess the focus of attention, participants performed a global-local letter task (Navon, 1977), in which they were presented with large letters composed of smaller letters and had to indicate as fast as possible which of the two target letters the display contained. Targets could be instantiated either by the large letter or by the small letters out of which the large letter was composed. In Experiments 1A and 1B, we reduced the intertrial interval (ITI) as compared to the original studies. The original ITI was 18–20 s. This rendered the experiment rather long and uneventful. Since motivation is a factor known to influence performance in online studies (e.g., Jun et al., 2017), the ITI was shortened to 6,000 ms in an attempt to keep the participants reasonably motivated. To see whether this had any impact on our findings, we later ran a second set of (otherwise identical) replications using the original ITIs (Experiments 2A and 2B, respectively).

## Experiments 1A and 1B

### Method

#### Participants

To ensure that our replication attempts were sufficiently powered, we chose to test more than twice the 32 participants tested by Gable and Harmon-Jones (2008, Experiment 2) and the same number of participants as Domachowska et al. (2016, Experiment 2) in all our experiments. More specifically, we accepted all participants who registered in the first wave of at least 80 registrations. Ninety-nine people participated in Experiment 1A (the replication of Gable & Harmon-Jones, 2008, Experiment 2). Participants who quit (11), participated in more than one of our experiments (5), or failed to achieve an accuracy above 65% (1) were excluded from the analysis. This applies to all the following experiments. Additionally, one did not perform the entire experiment due to an internet connection problem. Therefore, in the end, we analyzed data of 81 participants with a mean age of 27.48 years (*SD* = 9.35; range 18–63; 66 males; 14 females; 1 participant did not specify gender). Fifteen were native English speakers. There were 33 students, 37 workers, and 11 participants who did not specify their status. They received a reward of £4.16.

For Experiment 1B (the replication of Domachowska et al., 2016, Experiment 2), we wanted to have a sample size of at least 78 participants (as in the original study), we tested 84 participants. We excluded three participants who did not perform all the experimental blocks, one participant who participated in study 1A, and one who failed to achieve an accuracy above 65%. Therefore, our final example size was 79 participants, with a mean age of 27 years (*SD* = 8.97; range 18–58; 62 males; 14 females; 3 participants did not specify their gender). Seventeen of them were native English speakers. There were 37 students, 31 workers, and 10 participants who did not specify their status. They received a reward of £5.89.

Approval was obtained from the local ethics committee (CEP Reference No.: 2021-12-09-Bernhard Hommel-V2-3560 [Experiment 1A]; 2021-12-16-A. de Luca-V2-3596 [Experiment 1B]; 2022-01-26-Bernhard Hommel-V1-3694 [Experiment 2A]; 2022-01-26-Bernhard Hommel-V1-3696 [Experiment 2B]). All participants signed informed consent forms on Qualtrics, read the information sheet, agreed with each section of the consent form, and then continued with the experiment if they agreed to all consent requirements, and were naive about the purpose of the experiments. Furthermore, they reported being in good mental and physical health (this holds for all experiments reported here).

#### Apparatus

Open Sesame controlled stimulus presentation and data collection for all experiments and adapted the display to each participant's screen. To measure an individual's visual angle, it is necessary to present the target at the same viewing distance and to use the same display size. It is therefore impossible to measure an individual's visual angle without knowing its viewing distance and display size. Considering that the study was conducted online, we will not present the visual angles of the stimuli, but rather the OpenSesame codes. The food pictures (Blechert et al., 2014) were the same as those in Gable and Harmon-Jones (2008) and Domachowska et al. (2016) (see De Luca, 2022, Appendix A). All online tasks were recorded using a JATOS server (Just Another Tool for Online Studies; http://www.jatos.org/).

#### Experiment 1A

##### Global/Local Task and Affect Induction

To assess attentional breadth, we used the same Navon's global/local letter task (Navon, 1977) as the original studies of Gable and Harmon-Jones (2008) and Domachowska et al. (2016). The only change we made to our version of the Navon task was to use different letters. Our goal was to avoid any directional suggestions made by the stimuli. Therefore, symmetrical letters were chosen. Participants were presented with large letters made up of smaller letters. A target letter was presented in each trial, either a T or an H. The letters T and H required pressing the left and right control keys, respectively. S's and O's served as neutral stimuli. The target letters could appear either on the global level, with a T or H made of 10 local S's or 12 O's (5 per side and 2 at poles), or on the local level with a large S or O made of small 10 T's and H's (5 vertical and 5 horizontals, the font was Times New Roman). All stimuli were black and presented on a white background. The specific target was varied randomly and presented until a response or 5,000 ms, followed by another fixation cross (500 ms) that turned green (right) or red (error) during the training part. A global focus (broadened attentional breadth) was indicated by faster response to global targets, and faster responses to local targets indicated a local focus (narrowed attentional breadth). A picture of either a dessert to induce high motivational intensity, or a rock, to induce minimal motivational intensity preceded each trial. We used the same pictures as Gable and Harmon-Jones (2008).

##### Manipulation Check

After the Navon task, each picture used in the affect induction was presented again for 3 s, after which participants rated how pleasing (from very pleasing to very unpleasing) and arousing (from exciting to calm) it was using a 9-point Self-Assessment Manikin scales (Backs et al., 2005; Bradley & Lang, 1994). The desire for each pictured object was measured (1 = *really desired*, 9 = *did not desire*) on a numeric scale (see De Luca, 2022, Appendix A). Due to a program error, the scales of the questions were reversed in Experiments 1B and 2B. We recoded the reversed scales before analyses to maintain consistency.

##### Procedure

**Figure 1:**
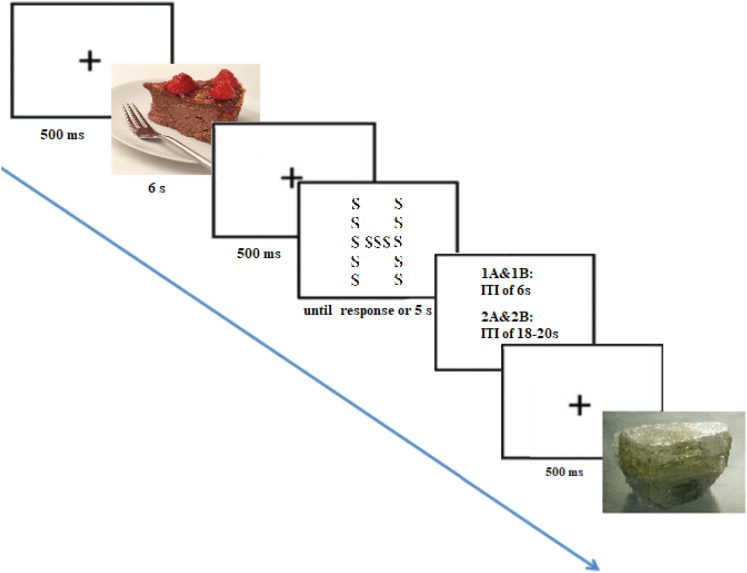
Prime and probe task stimuli in Experiments 1 and 2. A picture preceded each trial. The intertrial interval (ITI) lasted 6 s in Experiments 1A and 1B and 18–20 s, as in the original study, in Experiments 2A and 2B. We used the same pictures as Gable and Harmon-Jones (2008) and Domachowska et al. (2016). To assess attentional breadth, we used the same Navon's global/local letter task (Navon, 1977). Participants had to detect one of two letters (e.g., H or T), and each compound stimulus contained only one of the two letters, displayed at the local (small) or global (large) probe level. ITI = intertrial interval.

Participant recruitment was conducted online by the online platform Prolific. Data collection was conducted via Qualtrics (http://www.qualtrics.com/) and Open Sesame (Mathôt et al., 2011) through the JATOS server. As in Domachowska et al. (2016: Experiment 2), after obtaining informed consent, participants filled in the Positive and Negative Affect Schedule (PANAS) mood questionnaire (Watson et al., 1988) to control for possible confounding effects of the initial mood. Additionally, they carried out this questionnaire in Gable and Harmon-Jones' replication (2008). Participants then performed the Navon letter task with the affect induction. The procedure and timing of the Navon task were the same as in the original experiment, i.e., after six practice trials with neutral pictures, participants went through 64 experimental trials. Every trial began with a black fixation cross (500 ms; horizontal coordinates: *x*1 = −6 *x*2 = 6 *y*1 = 0 *y*2 = 0 *z*_index = 0; vertical coordinates: *x*1 = 0 *x*2 = 0 *y*1 = −6 *y*2 = 6 *z*_index = 0), followed by a picture (6,000 ms, OpenSesame codes: *draw image center = 1; file = “[foodImage]” scale = 1; show_if = always; x = 0 y = 0 z_index = 0*), another fixation cross (500 ms), and a letter from the Navon task (until response or 5,000 ms when no response was given; OpenSesame codes: *draw image center = 1; file = “[navonImage]”; scale = 1.5; show_if = always; x = 0 y = 0 z_index = 0*). The only difference from the original experiment was the ITI. To reduce boredom and the overall duration of the experiment, we replaced the original ITI of 18,000–20,000 ms with a shorter ITI of 6,000 ms (Figure 1). After the Navon task, the manipulation check was presented, whereafter participants were asked to indicate how long it had been since they had last eaten (in hours). After completing the experiment, participants were thanked, debriefed, and compensated.

#### Experiment 1B

The same procedure, software, and self-report measures to assess initial mood were used as in Experiment 1A. However, we now replicated the 3 (positive-high vs. positive-low vs. neutral) × 2 (local vs. global targets) within-participants design by Domachowska et al. (2016: Experiment 2). We used the same stimuli described in the original paper, i.e., for positive-high stimuli mouth-watering food pictures with high positive valence, high craving, and high palatability ratings, pictures of animals and flowers with high positive valence for positive-low stimuli, and for neutral stimuli, pictures of objects with lower valence. The list of the stimuli and their respective ratings as measured by us can be found in the supplemental materials.

##### Data Handling

Reaction times (RTs) were logarithmically transformed as in the original experiment. Practice trials, incorrect trials, and trials with RTs more than three *SD*s above the mean for the Navon task were excluded from the analysis (Fazio, 1990; Gable & Harmon-Jones, 2008).

Furthermore, we will present the percentages of errors (PEs) scores, which were not included in studies by Gable and Harmon-Jones (2008) and Domachowska and Domachowska (2016). The attentional breadth scores were calculated for RTs and percentages of errors (PEs) by subtracting RTs and PEs in the global trials from those in the local trials.

## Results

### Experiment 1A

#### Mood Check

We assessed the participants' baseline mood with the PANAS. We found no significant correlation between positive mood and the RT attentional breadth score in the dessert condition (*p* > .3) and the neutral condition (*p* = .058), nor did negative mood correlate with the RT attentional breadth score in the dessert condition or the neutral condition (*p*s> .2).

No significant correlation between positive mood and the PE attentional breadth score in any condition was found, *p*s> .4, nor did negative mood correlate with the PEs attentional breadth score in any condition, *p*s> .9.

#### Manipulation Check

A 2 (affective condition: positive-high [desserts] vs. neutral [rocks]) × 3 (ratings: valence vs. arousal vs. desire) repeated-measures ANOVA was conducted with participants' ratings of the pictures as the dependent variable. It revealed a significant interaction, *F*(2,160) = 5.86, η_*p*_^2^ = .068, *p* = .003. Participants rated the dessert pictures significantly more pleasing, arousing, and desirable than the rock pictures, all *p*s < .001 (see Table 1).

**Table 1:** Individual stimulus ratings as a function of affective condition

Rating	Affective condition					
	Positive-high		Positive-low		Neutral	
	*M*	*SD*	*M*	*SD*	*M*	*SD*
1A						
Valence	6.60a	1.31			4.72b	1.30
Arousal	5.78a	1.72			3.59b	1.22
Desire	6.11a	1.80			3.68b	1.37
1B						
Valence	7.12a	1.29	6.81b	1.12	6.85b	1.21
Arousal	6.04a	1.77	3.72b	2.21	3.66b	2.32
Desire	6.49a	1.48	3.16b	2.16	3.23b	2.22
2A						
Valence	6.57a	1.46			4.48b	1.33
Arousal	6.14a	1.58			3.78b	1.20
Desire	6.39a	1.70			3.69b	1.30
2B						
Valence	7.23a	1.19	7.05b	1.08	4.68c	1.13
Arousal	6.39a	1.63	4.39b	2.21		
Desire	6.64a	1.42	3.69b	2.47		
*Note*. Higher scores indicate higher ratings. Different subscripts between columns indicate that means differ at *p <* .05.						

#### Attentional Breadth

**Figure 2:**
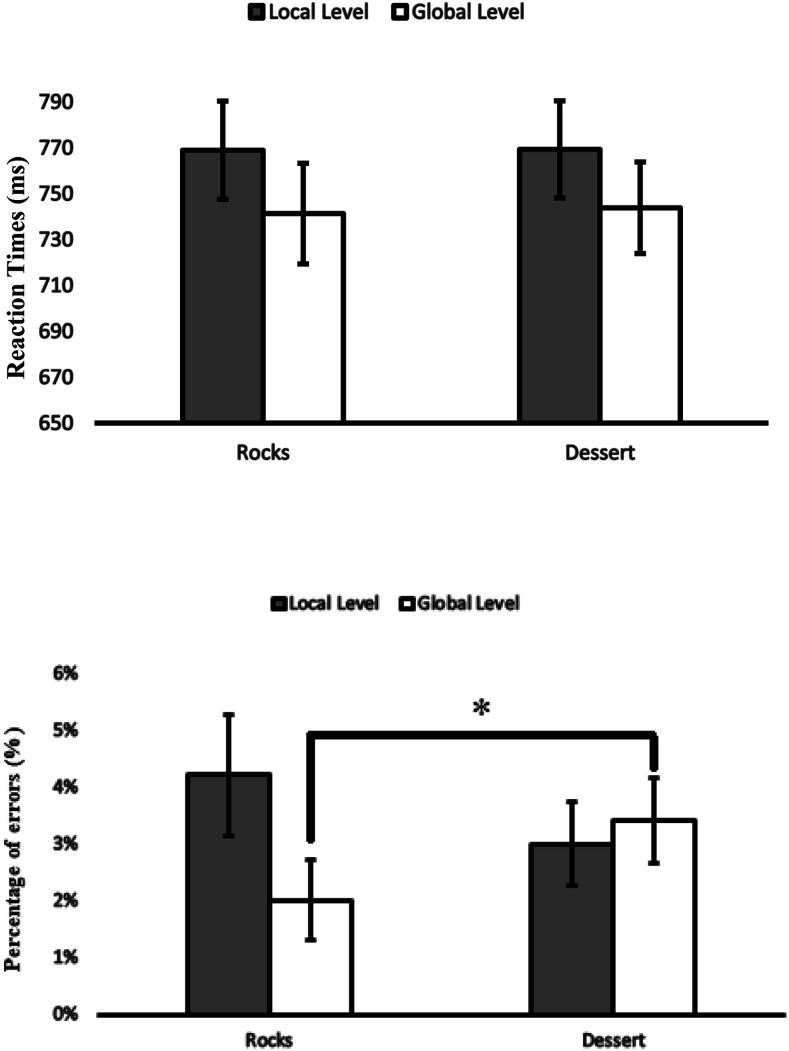
Experiment 1A. RTs and PEs in the Navon task for the local and global level as a function of affective condition. Error bars show standard errors. Asterisk indicates a significant affective condition effect on global level in PEs, *p* = .016.

Two 2 (affective condition: positive-high [desserts] vs. neutral [rocks]) × 2 (level: local vs. global) repeated-measures ANOVAs were conducted, one on RTs and one on PEs. They revealed a significant main effect of level on RTs, *F*(1,80) = 17.36, η_*p*_^2^ = .17, *p* < .001, but no main effect on PEs, *p* > .3, indicating that participants were faster but not more accurate when responding to the global level. We found no significant main effect of affective condition on RTs, *p* > .9, and PEs, *p* > .8. Importantly for our purpose, the interaction of affective condition with level on RTs, *p* > .8, was not significant. The PEs, however, yielded a significant interaction, *F*(1,80) = 8.00, η_*p*_^2^ = .09, *p* = .006. Separate ANOVAs showed that the affective condition was significant for the global level, *F*(1,80) = 6.05, η_*p*_^2^ = .07, *p* = .016, but not for the local level, *p =* .094. Participants made more errors on the global level after dessert pictures in comparison to the rock pictures (Figure 2). There were no other significant effects on RTs or PEs, *p*s> .38*.*

#### Time Last Eaten

We found no significant correlation between the time last eaten and the RT attentional breadth score in any affective conditions (*ps* > .3). Additionally, no significant correlations were found between the time last eaten and the PE attentional breadth score in any affective conditions (*ps* > .4).

#### Bayesian Analysis

We performed a Bayesian repeated-measures ANOVA to compare the entire model to a model excluding the Level × Affective condition. For fixed effects, a prior scale of 0.5 was used. For RTs, the model without the interaction effect was 5.85 times more favored than the model including the interaction, corresponding to substantial evidence against an interaction effect. For PEs, the model without the interaction effect was 1.2 times more favored than the model, including the interaction, suggesting insufficient statistical power (Jeffreys, 1998). Moreover, we reran the experiment (Experiment 2B), increasing the ITI, and we gained sufficient power.

### Experiment 1B

#### Mood Check

We found no significant correlation between positive mood and the RT attentional breadth score in any affective conditions, *p*s> .3. Also, negative mood did not correlate with the RT attentional breadth score in any affective conditions, *p*s> .3. Additionally, no significant correlation between positive mood and PE attentional breadth score in any of the affective conditions, *p*s> .4, was found. Negative mood also did not correlate with PEs attentional breadth score in the positive-high condition (*p* = .068) or in other affective conditions, *p*s> .6.

#### Manipulation Check

A 3 (ratings: valence vs. arousal vs. desire) × 3 (affective condition: positive-high vs. positive-low vs. neutral) repeated-measures ANOVA was conducted. Participants' ratings of the pictures as the dependent variable revealed a significant interaction effect, *F*(4,308) = 80.52, η_*p*_^2^ = .51, *p* < .001. Positive-high pictures differed significantly from positive-low pictures in arousal, desirability, and in valence, *F*(2,154) = 91.69, η_*p*_^2^ = .54, *p* < .001. Additionally, positive-high pictures differed significantly from neutral pictures in arousal, desirability, and valence, *F*(2,154) = 85.76, η_*p*_^2^ = .52, *p* < .001 (see Table 1). Importantly, positive-low pictures did not differ from neutral pictures in arousal, desirability, and valence, *p* > .4.

#### Attentional Breadth

**Figure 3:**
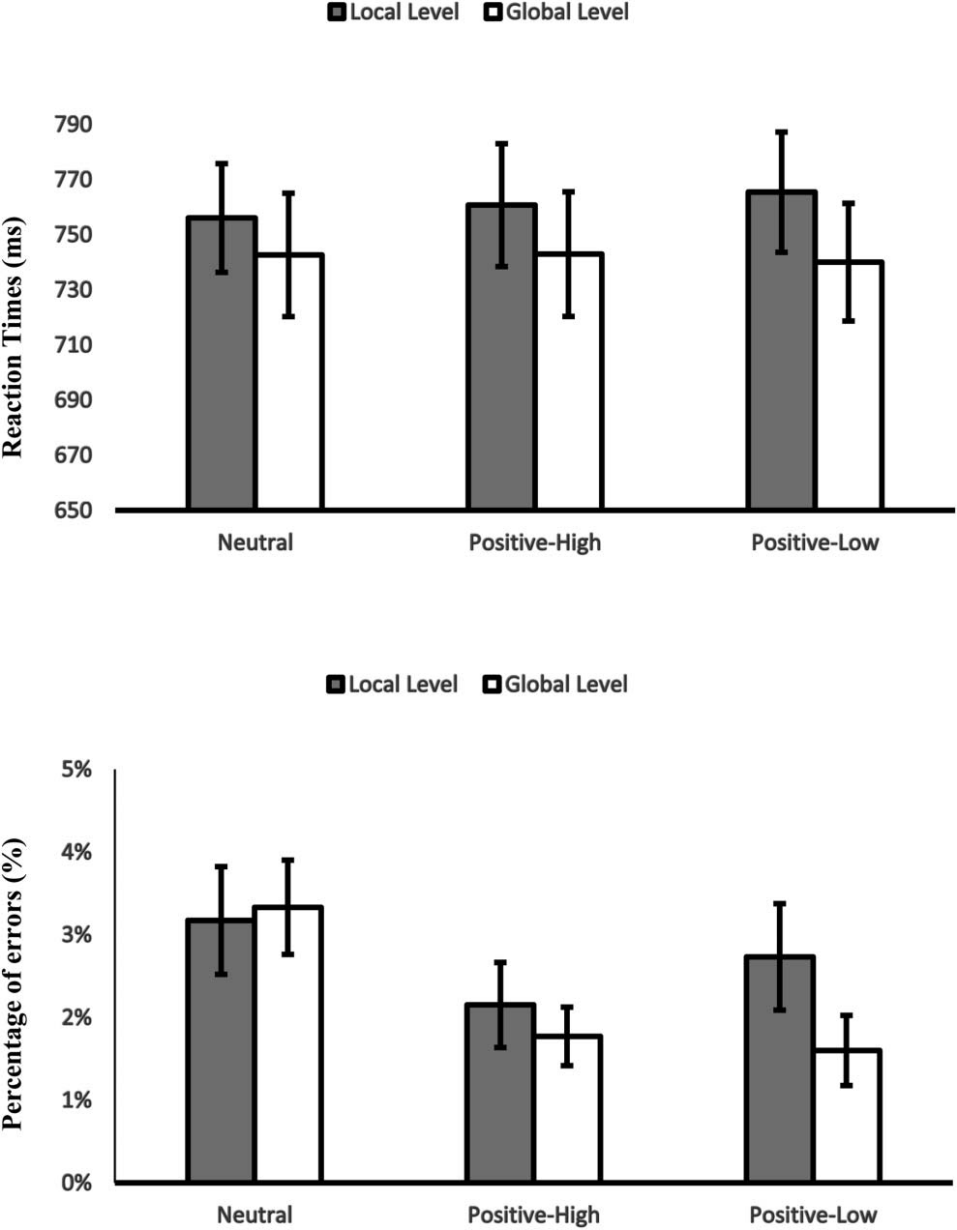
Experiment 1B. RTs and PEs in the Navon task for the local and global level as a function of affective condition. Error bars show standard errors.

Two 2 (level: local vs. global) × 3 (affective condition: positive-high vs. positive-low vs. neutral) repeated-measures ANOVAs were conducted, one for RTs and one for PEs. They revealed a significant main effect of level on RTs, *F*(1,78) = 14.07, η_*p*_^2^ = .15, *p* < .001, but no for PEs, *p* > .3, indicating that participants were faster but not more accurate when responding to global level. No significant effect of affective condition on RTs was found (*p* > .8), but the analysis showed a significant effect of affective condition on PEs, *F*(2,156) = 5.20, η_*p*_^2^ = .063, *p* = .006. A separate ANOVA showed that participants made more errors after neutral pictures in comparison to positive-high pictures, *F*(1,78) = 8.38, η_*p*_^2^ = .097, *p* = .005, and to positive-low pictures, *F*(1,78) = 5.24, η_*p*_^2^ = .063, *p* = .025. Importantly, we found no interaction of affective condition with level on RTs, *p* > .7, and on PEs, *p* > .3 (Figure 3). No other significant effects on RTs and PEs were found, *p*s > .3.

#### Time Last Eaten

We found no significant correlation between the time last eaten and RT attentional breadth score in the positive-high condition, *p* = .065, nor did they correlate in the positive-low condition and neutral condition, *p*s> .8. Time last eaten did not correlate with PE attentional breadth score in any affective conditions, *p*s> .1.

#### Bayesian Analysis

We also performed a Bayesian repeated-measures ANOVA to compare the entire model to a model excluding the Level × Affective condition. For fixed effects, a prior scale of 0.5 was used. For RTs, the model without the interaction effect was 14.54 times more favored than the model including the interaction corresponding to strong evidence against an interaction effect. On PEs, the model without the interaction was 8.33 times more favored than the model including the interaction corresponding to substantial evidence against an interaction effect (Jeffreys, 1998).

## Discussion Experiments 1A and 1B

Both experiments replicated the well-known basic global precedence effect (Navon, 1977), and our manipulation checks show that the manipulations of affect and motivation were successful. Yet, none of the experiments replicated the interaction effect of attentional breath with affective condition on RTs reported in the replicated studies. While we found a significant (but underpowered) effect in the expected direction in the error rates of Experiment 1A (which was not reported in Gable & Harmon-Jones, 2008), no such effects were found for RTs in Experiment 1A and 1B, nor on the error rates of Experiment 1B. However, before jumping to conclusions, we considered the possibility that our decision to change the original ITI parameter might have affected the outcome. While it would be odd and theoretically hard to explain how this parameter would make such a decisive difference, it was the only substantial change from the original studies besides them being online and the different letters in the Navon task. To exclude that the new ITI accounts for our failure to replicate, we thus reran both experiments with the original ITI. Furthermore, there is no significant correlation between baseline positive or negative mood and RT attentional breadth in any of the affective conditions, which contradicts the GLOMOsys theory of mood impacting global and local processing.

## Experiments 2A and 2B

### Method

#### Participants

For Experiment 2A, we tested 89 participants. Five quit the study, two already participated in Experiment 1B, and 2 failed to achieve an accuracy above 65%. They were excluded from the analysis. Therefore, our final sample size was 80 participants with a mean age of 29.75 years (*SD* = 9.02; range 19–60; 50 males; 30 females), of whom 37 were native English speakers. There were 31 students, 40 workers, and 9 participants who did not specify their status. They received a reward of £6.88.

Ninety participants participated in Experiment 2B; 10 were excluded from the analysis because they quit the experiment. Thus, we analyzed data from 80 participants with a mean age of 26.93 years (*SD* = 9.01; range 19–60; 51 males; 29 females). Twenty of them were native English speakers. There were 45 students, 29 workers, and 6 participants who did not specify their status. They received a reward of £9.38.

##### Procedure

Everything was the same as in Experiments 1A and 1B, except for the ITI, which was increased to 18,000–20,000 ms as in the original experiment***.***

## Results

### Experiment 2A

#### Mood Check

We found no significant correlation between baseline positive mood and the RT attentional breadth score in any of the affective conditions, *ps* > .9. Also, negative mood did not correlate with the RT attentional breadth score in any of the affective conditions, *p*s> .3. There was no significant correlation between positive mood and PE attentional breadth score in any of the affective conditions, *ps* > .1. Also, negative mood did not correlate with the PEs attentional breadth score in the dessert condition, *p* > .6, and in the neutral condition, *p* = .055.

#### Manipulation Check

A repeated-measures ANOVA was conducted with 2 (affective condition: positive-high [desserts] vs. Neutral [rocks]) × 3 (ratings: valence vs. arousal vs. desire). Participants' ratings of the pictures as the dependent variable revealed a significant interaction, *F*(2,158) = 8.17, η_*p*_^2^ = .094, *p* < .001. Participants rated the dessert pictures significantly more pleasing, arousing, and desirable than the rock pictures, all *ps* < .001 (see Table 1).

#### Attentional Breadth

**Figure 4:**
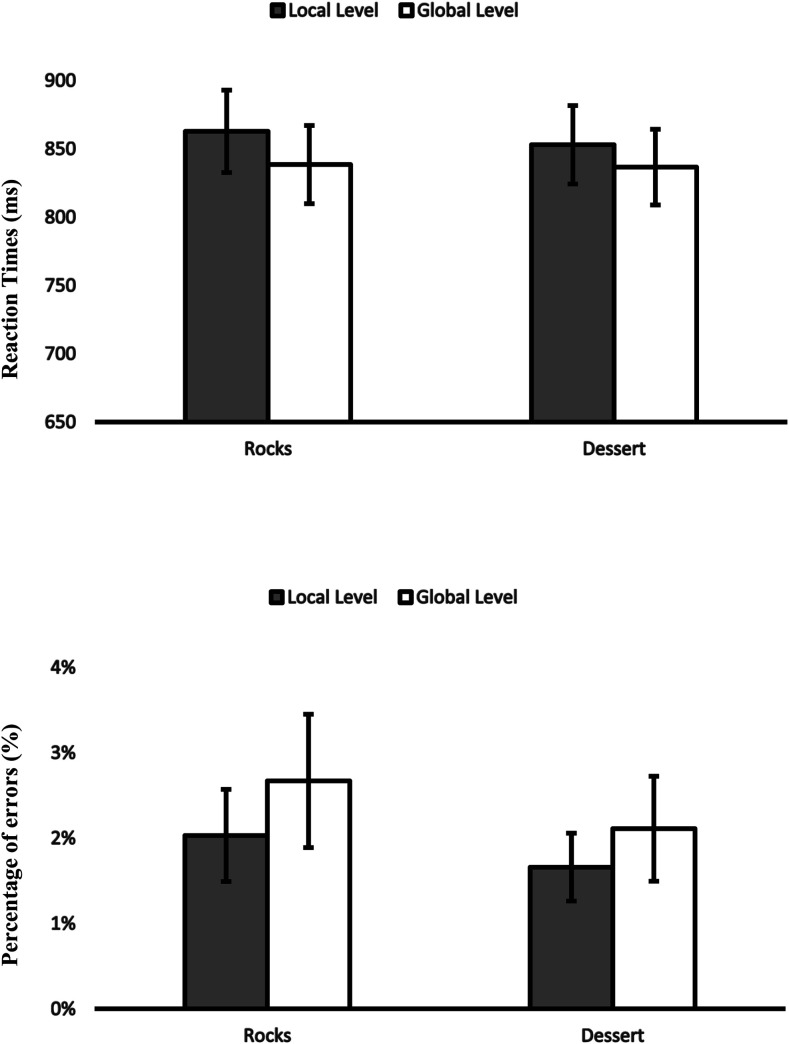
Experiment 2A. RTs and PEs in the Navon task for the local and global level as a function of affective condition. Error bars show standard errors.

Two 2 (affective condition: positive-high [desserts] vs. Neutral [rocks]) × 2 (level: local vs. global) repeated-measures ANOVAs were conducted, one for RTs and one for PEs. The repeated-measures ANOVAs revealed a significant main effect of level on RTs, *F*(1,79) = 5.27, η_*p*_^2^ = .063, *p* = .024, but no main effect of level on PEs, *p* > .2, indicating that participants were faster when responding to global level. Notably, the analysis showed no significant effect of affective condition on RTs, *p* > .6, and PEs, *p* > .2, and no interaction with level in RTs, *p* > .5, and in PEs, *p* > .8 (Figure 4).

#### Time Last Eaten

We found no significant correlations between the time last eaten and an RT attentional breadth score in any of the affective conditions, *ps* > .1. Additionally, time last eaten and PEs attentional breadth score did not correlate in any affective conditions, *ps* > .1.

#### Bayesian Analysis

A Bayesian repeated-measures ANOVA was employed to compare the entire model to a model excluding the Level× Affective condition. For fixed effects, a prior scale of 0.5 was used. On RTs, the model without the interaction effect was 5.18 times more favored than the model including the interaction, and also on PEs, it was 5.83 times more supported than the model including the interaction corresponding to substantial evidence against any interaction effect (Jeffreys, 1998).

### Experiment 2B

#### Mood Check

No significant correlation between positive mood score and the RT attentional breadth score was found in any affective conditions, *ps* > .3. However, they did correlate in the positive-low condition, *r* = .28, *p* = .009, meaning that participants with higher positive mood showed a higher RT attentional breadth score in the positive-low condition. Negative mood scores did not correlate with the RT attentional breadth scores in any affective condition (*p*s> .3). We found no significant correlation between positive mood scores and PE attentional breadth score in any affective condition (*p*s> .1), nor correlations between PEs attentional breadth score and negative mood in any affective condition (*p*s> .4).

#### Manipulation Check

We initially planned to assess participants' ratings of the pictures with the three scales (valence vs. arousal vs. desire) as in Experiment 1B. However, participants did not rate the arousal and desire scale for the neutral pictures but only the valence due to a programming error. Therefore, we ran a 3 (ratings: valence vs. arousal vs. desire) × 2 (affective condition: positive-high vs. positive-low) repeated-measures ANOVA with participants' ratings of the pictures as the dependent variable. Positive-high pictures differed significantly from positive-low pictures in valence, arousal, and desire, *F*(2,158) = 71.96, η_*p*_^2^ = .47, *p* < .001 (see Table 1). Participants rated the positive-high pictures as significantly more pleasing, arousing, and desirable than the positive-low pictures, all *ps* < .001 (see Table 1).

Then, we conducted a 3-way (affective condition: positive-high vs. positive-low vs. neutral) ANOVA with participants' valence ratings of the pictures. The analysis showed positive-high and positive-low pictures differed significantly from neutral pictures in valence, *F*(2,158) = 232.23, η_*p*_^2^ = .74, *p* < .001 (see Table 1). Participants rated the positive-high pictures as significantly more pleasing than the neutral pictures, *F*(1,79) = 299.55, η_*p*_^2^ = .79, *p* < .001, and participants rated the positive-low pictures also more pleasing than the neutral pictures, *F*(1,79) = 300.20, η_*p*_^2^ = .79, *p* < .001, all *ps* < .001 (see Table 1).

#### Attentional Breadth

Two 2 (level: local vs. global) × 3 (affective condition: positive-high vs. positive-low vs. neutral) repeated-measures ANOVAs were conducted, one for RTs and one for PEs. They revealed a significant main effect of level on RTs, *F*(1,79) = 8.05, η_*p*_^2^ = .093, *p* = .006, but no main effect of level on PEs, *p* > .5, indicating that participants were faster but not more accurate when responding to global level.

However, we found a significant effect of affective condition on RTs, *F*(2,158) = 5.48, η_*p*_^2^ = .065, *p* = .005. Further analysis showed that participants were faster on the Navon task after low-positive pictures in comparison to high-positive pictures RTs, *F*(179) = 7.91, η_*p*_^2^ = .091, *p* = .006, and in comparison to neutral pictures RTs, *F*(1,79) = 7.36, η_*p*_^2^ = .085, *p* = .008. There was no effect of affective condition on PEs, *p* = .080.

**Figure 5:**
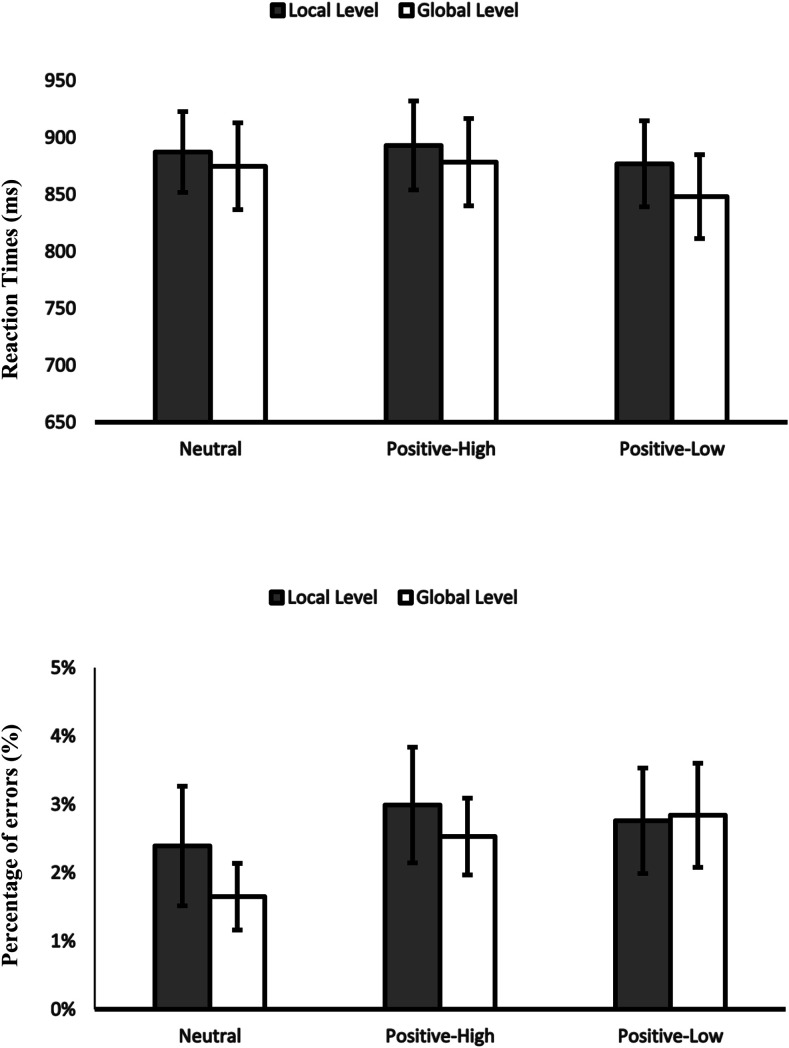
Experiment 2B. RTs and PEs in the Navon task for the local and global level as a function of affective condition. Error bars show standard errors.

More importantly for our research, no interaction of affective condition with level on RTs, *p* > .6, and on PEs, *p* > .5, was found (Figure 5). There was no other significant effect in RTs or PEs, *p*s > .59*.*

#### Time Last Eaten

We found no significant correlation between the time last eaten and the RT attentional breadth score in any affective condition (*p*s > .5), nor any correlations between PEs attentional breadth score and the time last eaten in any affective condition (*p*s > .3).

#### Bayesian Analysis

Finally, we performed a Bayesian repeated-measures ANOVA to compare the full model to a model excluding the level*affective condition. For fixed effects, a prior scale of 0.5 was used. For RTs, the model without the interaction effect was 14.17 times more favored than the model including the interaction; for PEs, it was 17.91 times. This corresponds to strong evidence against any interaction effect (Jeffreys, 1998).

## Discussion Experiments 2A and 2B

Although our manipulation checks showed that our manipulations were effective and Experiments 2A and 2B showed the basic global precedence effect and used the ITIs of the original experiments, our results show no interaction between the affective condition and attentional breadth. This means we again failed to replicate the main results of Gable and Harmon-Jones (2008, Experiment 2) and Domachowska et al. (2016, Experiments 1 and 2).

Additionally, no significant correlation has been found between baseline positive or negative mood and attentional breadth in any of the affective conditions except in the positive-low condition in Experiment 2B, which contradicts part of the GLOMOsys theory of mood impacting global and local processing.

## Exploratory Analysis

As a result of the design similarities, we also conducted a cumulating meta-analysis (Braver et al., 2014; Goh et al., 2016), combining the data from all 4 experiments, and disregarding the “positive/low conditions” from Experiments 1B and 2B.

### Attentional Breadth

**Figure 6:**
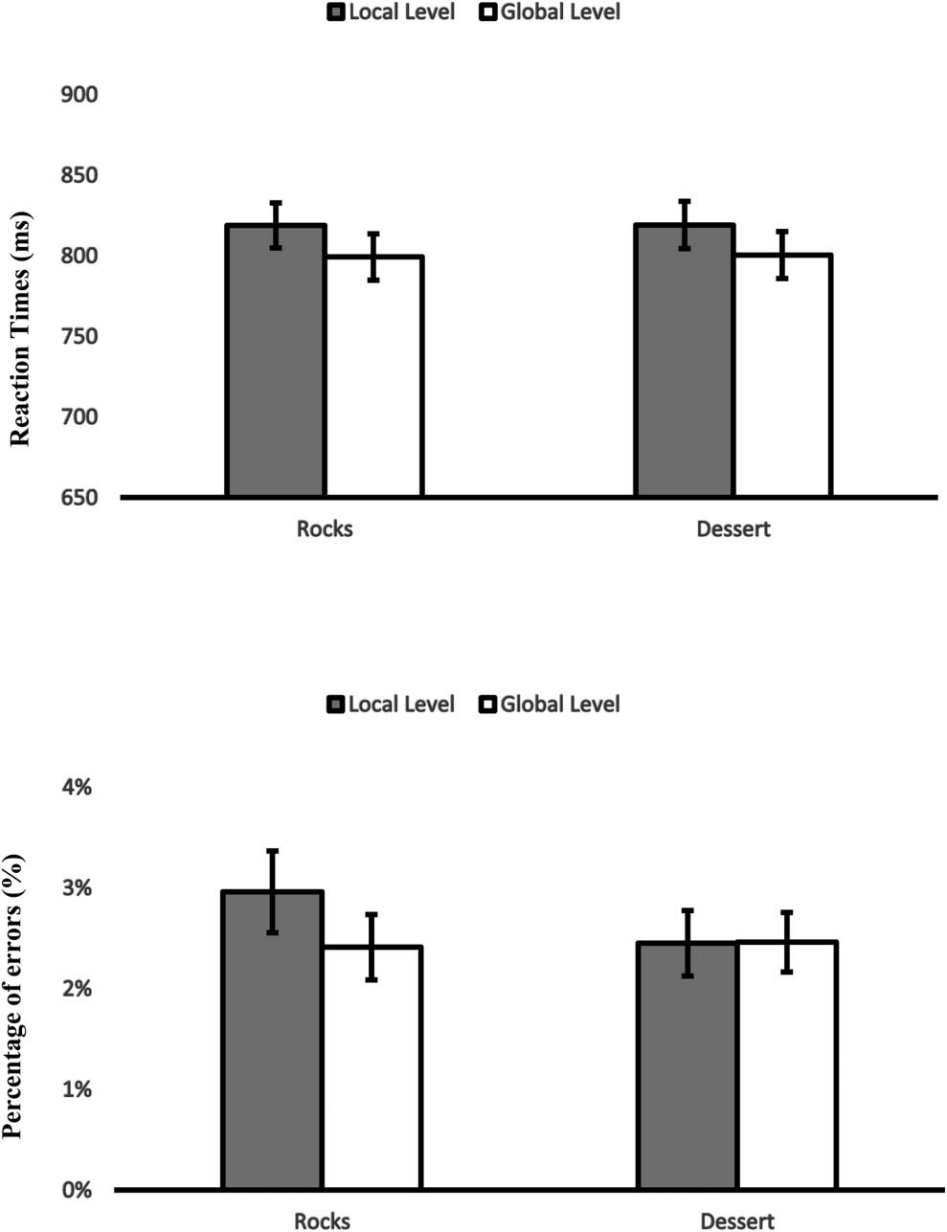
Meta-analysis of all 4 experiments. RTs and PEs in the Navon task for the local and global level as a function of affective condition. Error bars show standard errors.

Two 2 (affective condition: positive-high [desserts] vs. neutral [rocks]) × 2 (level: local vs. global), experiment (1A, 1B, 2A, 2B) as between-participant factors, repeated-measures ANOVAs were conducted, one on RTs and one on PEs. They revealed a significant main effect of level on RTs, *F*(1,316) = 31.37, η_*p*_^2^ = .09, *p* < .001, but no main effect on PEs, *p* > .4, indicating that participants were faster but not more accurate when responding to the global level. We found no significant main effect of affective condition on RTs, *p* > .8, and PEs, *p* > .2. Importantly for our purpose, the interaction of affective condition with level was not significant on RTs, *p* > .7, or PEs, *p* > .1 (Figure 6)*.*

### Conclusion

The present study aimed to verify claims regarding the effects of positive mood and/or motivational intensity (Domachowska et al., 2016; Gable & Harmon-Jones, 2008) on attentional breadth as assessed by Navon's global/local task (Navon, 1977). The original studies (Gable & Harmon-Jones, 2008, Experiment 2; Domachowska et al., 2016, Experiment 2) observed that positive stimuli associated with high approach motivation reduced attentional breadth in a Navon task as compared to control stimuli. In four well-powered experiments and with close checks of the affective qualities of our stimulus material, we failed to replicate this effect. Further analysis of our data failed to indicate a linear relationship between attentional breadth and desirability of stimuli (and, by inference, motivational intensity). Then, we computed a correlation between subjective desire ratings (as a proxy for motivational intensity) and attentional breadth across participants, which however remained nonsignificant even after controlling for group-wise effects of the following stimuli (positive-high, positive-low, neutral).

Furthermore, since there were similar design aspects between the experiments, we also conducted an exploratory analysis, using the data from each experiment, but disregarding the positive/low conditions from Experiments 1B and 2B. However, attentional breadth and effective condition did not interact.

As a result, valence, arousal, and desirability did not seem to be statistically significant mediators of attentional breadth, although there was a difference in the desirability of the three types of stimuli. Neither desirability nor attentional breadth was affected by the time since the last meal. Since participants reported the time they last ate in hours, it might have been too coarse-grained to detect more subtle influences.

Moreover, we found that baseline positive/negative mood does not correlate significantly with attentional breadth in any of the affective conditions, except for the positive low condition in Experiment 2B. Considering this, we can say the mood check analysis contradicts GLOMOsys' theory regarding mood influencing global and local processing. Unfortunately, we are unable to compare our data with Gable and Harmon-Jones (2008) because they did not use a mood check, and Domachowska et al. (2016) did not have the opportunity to analyze those due to a program error.

More specifically, our results showed no significant difference between the conditions with high and low motivational intensity when processing local information, suggesting that positive affect does not broaden the attentional focus. Our failures to replicate in Experiments 1A and 1B might be due to the different ITI we used. However, Experiment 2, which did use the original ITIs, also failed to replicate the original findings.

Furthermore, we did not use the exact same letters for the Navon task. Although this could be argued to make a difference (which would be very hard to theoretically motivate, however) and render our replication *conceptual* instead of a *direct* replication, we did replicate the global precedence effect in all studies. Indeed, since the responses were defined in terms of horizontal location, and as directional letters have been shown to affect the spatial location of visual attention (Hommel, 1995), we are confident that using nondirectional letters is actually a better choice. For all intents and purposes, the global precedence effect shows our Navon task was effective.

Additionally, one might argue that the online nature of our experiments caused the failure to replicate the original studies. Indeed, some have argued that the laboratory setting results in more reliable results (Birnbaum, 2004; Chandler et al., 2014; Dandurand et al., 2008). However, by now, there have been many online replications of the more robust cognitive effects (Germine et al., 2012; Logie & Maylor, 2009). Nevertheless, it is worth noting that in an online session, experimental conditions are not as controlled as in a laboratory, which may contribute to online replication failures (Fortenbaugh et al., 2015; Ralph et al., 2014; Sadeh et al., 2011; Thomson et al., 2016). Although this might be true for other studies, we think this is not the case here. Our main argument is that our online setup was sensitive enough to reliably measure the basic global precedence effect and we were able to measure differences in valence during the manipulation checks. These results imply that our manipulations were successful and overall task motivation cannot have been sub-par. Furthermore, Peer et al. (2021) observed that participants on Prolific paid more attention to questions, understood instructions better, and reacted more honestly (despite the opportunity to cheat) as compared to other platforms. Additionally, Peer et al. (2017) found that Prolific samples generate high-quality data and are highly reliable.

As opposed to the laboratory experiments of Gable and Harmon-Jones (2008, Experiment 2) and Domachowska et al. (2016, Experiments 1 and 2), which included university students from a limited range of demographics, our online replication produced a relatively large variability in demographics, robust random sampling (since participants self-enroll at their convenience), and large sample size. Although Domachowska et al. (2016) concluded from their results that the effects of motivation on attention were robust across cultures, our results make this less likely.

Taken altogether, our repeated failures to replicate cast serious doubt on the claim that mood or motivational attitude has a systematic impact on the processing of global versus local visual information.

## Notes

**Publication Ethics:** Approval was obtained from the local ethics committee (CEP Reference No.: 2021-12-09-Bernhard Hommel-V2-3560 [Experiment 1A]; 2021-12-16-A. de Luca-V2-3596 [Experiment 1B]; 2022-01-26-Bernhard Hommel-V1-3694 [Experiment 2A]; 2022-01-26-Bernhard Hommel-V1-3696 [Experiment 2B]).

**Open Data:** Our design and confirmatory analyses were pre-registered on the Open Science Framework (OSF; Gable replication: https://osf.io/et3am/; Domachowska replication: https://osf.io/4q6hy).

Supplementary data (Appendix A) to this article can be found online at https://osf.io/skpyc/?view_only=cf3a2812304f4632af44d45d22d5f904 (De Luca, 2022).
